# An automatic parathyroid recognition and segmentation model based on deep learning of near‐infrared autofluorescence imaging

**DOI:** 10.1002/cam4.7065

**Published:** 2024-03-08

**Authors:** Fan Yu, Tian Sang, Jie Kang, Xianzhao Deng, Bomin Guo, Hangzhou Yang, Xiaoyi Chen, Youben Fan, Xuehai Ding, Bo Wu

**Affiliations:** ^1^ Department of Thyroid Breast and Hernia Surgery, Shanghai Sixth People's Hospital Affiliated to Shanghai Jiao Tong University School of Medicine Shanghai China; ^2^ School of Computer Engineering and Science Shanghai University Shanghai China; ^3^ Ningbo Institute of Life and Health Industry University of Chinese Academy of Sciences Ningbo China

**Keywords:** artificial intelligence, medical segmentation, near‐infrared autofluorescence imaging, parathyroid gland, thyroidectomy

## Abstract

**Introduction:**

Near‐infrared autofluorescence imaging (NIFI) can be used to identify parathyroid gland (PG) during surgery. The purpose of the study is to establish a new model, help surgeons better identify, and protect PGs.

**Methods:**

Five hundred and twenty three NIFI images were selected. The PGs were recorded by NIFI and marked with artificial intelligence (AI) model. The recognition rate for PGs was calculated. Analyze the differences between surgeons of different years of experience and AI recognition, and evaluate the diagnostic and therapeutic efficacy of AI model.

**Results:**

Our model achieved 83.5% precision and 57.8% recall in the internal validation set. The visual recognition rate of AI model was 85.2% and 82.4% on internal and external sets. The PG recognition rate of AI model is higher than that of junior surgeons (*p* < 0.05).

**Conclusions:**

This AI model will help surgeons identify PGs, and develop their learning ability and self‐confidence.

## 1 INTRODUCTION

The identification of PGs is the important role in thyroidectomy and parathyroidectomy. The similarity of PG, lymph node, and adipose tissue increases the difficulty of identification. If unfamiliar with the morphology and anatomy of PG, it is highly susceptible to be damaged, leading to hypocalcemia which affects the quality of patient's life. It is widely accepted that the incidence of temporary and permanent hypoparathyroidism after operation is 5.94% ~ 67.69% and 0 ~ 20%.^1^ NIFI is a noninvasive and specific method of intraoperative PGs recognition. Clinical researches have confirmed that NIFI can identify more PGs than naked eye.^2^, ^3^, ^4^ Some follow‐up studies found that the adjunctive detection of NIFI is helpful to improve the early postoperative hypocalcemia.^5^, ^6^ By virtue of the NIFI, it is able to distinguish PGs from other tissues. However, it has been found that PGs with autofluorescence intensity and show different brightness on the image.^7^ In addition, other tissues in the surgical field, such as lymph node, adipose tissue and thyroid nodule, can present bright fluorescent signal that interfere with the surgeon's judgment of PGs. Even experienced surgeons lack sufficient evidence to clarify which one is the true PG and the histopathology or puncture verification is necessary, which increases operation time while damaging part of PG, and affecting surgeon confidence. Therefore, the actual prognostic value of NIFI still needs to be further explored, but it is undeniable that more and more surgeons using it to help identify PGs during operation.

In recent years, deep learning, especially convolutional neural networks, has rapidly developed into a research hotspot in medical image analysis. At present, the research frontier of the combination of deep learning and medical fields covers many fields including image classification, detection and segmentation, image registration, etc. In 2015, Olaf Ronneberger and others proposed the U‐Net network^8^ to solve the problem of small amount of medical image data and high labeling costs. The U‐Net network can complete image segmentation tasks with high accuracy on small‐scale data, which provides a good idea for the application of deep learning in the medical field. With the success of U‐Net in medical image segmentation, a series of U‐Net networks have been applied to medical segmentation. For example, V‐Net is used in the segmentation of 3D medical images in prostate MRI image segmentation.^9^ Sha et al. applied the U‐Net series of models for RGB images segmentation task.^10^ These methods confirmed that AI model could assist surgeons in judging lesions or content of concern to a certain extent by providing surgeons with the possible position and shape of PGs, which may reduce the interference of false positives in PGs identification.

The segmentation task in computer science is to use AI methods such as neural networks to predict the outline of the object from the image. This task is very suitable for marking the lesion area in medicine. Among the segmentation models, Mask‐RCNN^11^ is a classic instance segmentation model. Many subsequent methods are proposed based on the Mask‐RCNN architecture, including Sparse‐RCNN. With the introduction of the Transformer architecture^12^ into the field of Computer Vision, ISTR^13^ is a segmentation network based on the Transformer architecture. In this study, the differences between the three models selected in the segmentation part were compared, and ISTR model achieved highest result. So ISTR was selected as the segmentation model in this method.

The purpose of this study is to put forward a novel deep learning method to help identify PGs under NIFI, reduce the interference of false positive tissues in NIFI to surgeons, and improve the recognition accuracy.

## 2 METHODS

Between August 2021 and October 2022, 103 patients who underwent thyroidectomy and parathyroidectomy at Shanghai Sixth People's Hospital. This study was approved by the human subjects ethics board of Shanghai Sixth People's Hospital (Approval No: 2022‐KY‐178 (K)) and was conducted in accordance with the Helsinki Declaration of 1975, as revised in 2013. The written informed consent statements were obtained from all patients in this study.

**FIGURE 1:**
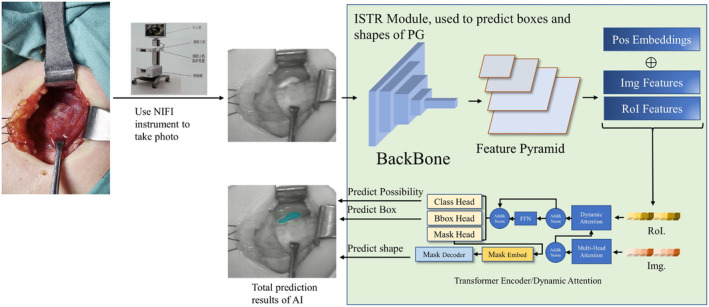
AI model of parathyroid detection and segmentation method.

The NIFI device (SHINEVIA, China) used during surgery consists of a NIFI camera, a computer system and a display. The camera was placed at 15 cm from surgical field and the autofluorescence images were presented on display. After storing NIFI images, the PGs in these images were confirmed and labeled by a specialized surgeon. Subsequently, 452 pictures of 79 patients collected from August 24, 2021 to July 29, 2022 were randomly divided into a training set and an internal verification set according to a ratio of 8:2. The training set was used to train the AI model, while the validation set was used for testing the performance of our AI model. The specific method flow is shown in the Figure 1.

### 2.1 Surgical technique

The assistant used the NIFI camera 15 cm away from the surgical field after dissection of the thyroid membrane (Stage I), after removal of thyroid gland (Stage II) and isolated tissues (Stage III) when thyroidectomy. Stage I and Stage III were detected when parathyroidectomy. Before identifying PGs, white lights needed to be turned off to reduce the intake of unnecessary light causing interference. PG was determined visually by a surgeon with more than 10 years of experience in thyroid surgery. Each PG was verified by rapid measurement of parathyroid hormone (PTH) levels by immune colloidal gold technique (ICGT). Isolated tissues was eventually verified by histopathology for the possibility of miscutted PG. All images was stored.

### 2.2 Data analysis

**FIGURE 2:**
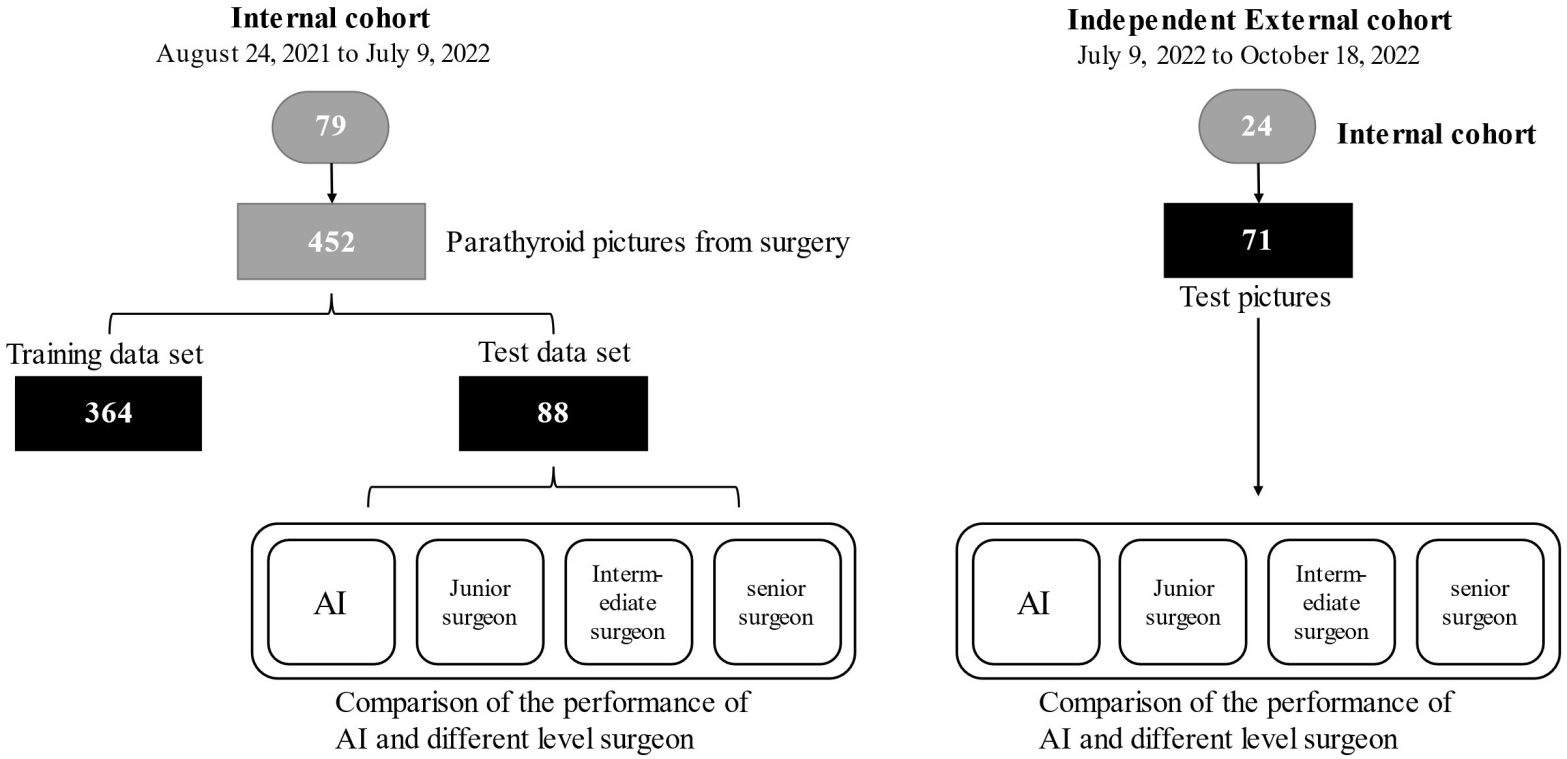
Composition of our dataset. The flowchart shows the establishment of our method, and comparison of our method among junior surgeon, intermediate surgeon and senior surgeon. Every parathyroid was token 2–4 images.

In this study, the NIFI image was used as the object of instance segmentation network recognition to assist doctors in identifying NIFI. Five hundred and twenty three NIFI images with clear PGs presence in 103 patients were used as the data set source for the objects identified by the instance segmentation network. Among them, 79 patients were divided into training set and internal validation set with a ratio of 8:2. Another professional surgeon used labelme software to label the confirmed parathyroid glands in NIFI images. Each PG in the images is validated using ICGT, known for its higher diagnostic accuracy compared to frozen section examination. The latter can be altered by fine needle aspiration (FNA) with rapid PTH determination.^14^ Segmentation techniques predict PG shapes and contours, aiding tissue preservation and enhancing visualization. In addition, the NIFI images of 24 patients undergoing thyroidectomy or parathyroidectomy from July 9, 2022 to October 18, 2022 were collected as an independent external validation cohort. We performed preprocessing operations on the data to remove images such as blurred images and inaccurate focus to obtain the input data. The detailed flowchart of our method establishment and analysis is shown in Figure 2.

The number of PGs identified under white light and NIFI at each stage in a cohort of 103 patients was recorded. Meanwhile, sensitivity, specificity and accuracy of NIFI were calculated to evaluate the efficiency of NIFI technique in the identification of PGs.

35 of 103 patients who underwent total thyroidectomy were examined for PTH levels (normal reference range 15–65 pg/mL) before surgery, postoperative day 1, 1 month, 3 months, and 6 months. The occurrence of symptomatic hypocalcemia (hand and foot twitching, limb numbness) was also recorded.

### 2.3 AI model

In this study, we used ISTR network as the AI model, which was implemented by Python and PyTorch. The training set was used as the training data for the training process of the model, and the test set was used to evaluate the performance of the model. The indicators evaluated by this model were divided into instance segmentation indicators and medical indicators, and the sensitivity and specificity of medical indicators and NIFI intraoperative detection of PGs were the same. The instance segmentation evaluation metrics used the COCO evaluation metrics.^15^ In detail, prediction results with Intersection over Union (IoU) with the ground truth more than 50% and confidence score more than 0.5 were evaluated as positive samples for statistical analysis. After training, the image data of the test set was input to the model to evaluate the performance of the AI model. The model could provide the location of possible PGs and mask its specific outline and give a confidence score. We calculated their precision and recall according to the following formulas. Among them, TP (True Positive) represents true positive samples, FP (False Positive) represents false positive samples, TN (True Negative) represents true negative samples, and FN (False Negative) represents false negative samples.

$$\text{Precision}=\mathrm{TP}/\left(\mathrm{TP}+\mathrm{FP}\right)$$

$$\text{Recall}=\mathrm{TP}/\left(\mathrm{TP}+\mathrm{FN}\right)$$

### 2.4 Image verification

The result of AI model recognition was visualized and analyzed to calculate the recognition rate of PGs. The original images from the validation set were selected and PGs in the images were identified and depicted by a senior surgeon (more than 20 years of experience in thyroid surgery), an intermediate surgeon (more than 10 years of experience in thyroid surgery) and a junior surgeon (2 years of experience in thyroid surgery). A chi‐square test was used to compare the PGs recognition rate between AI model and different group of surgeons. Comparisons between groups were statistically processed by SPSS 26.0, and statistical significance was assigned for *p* values <0.05. Continuous data were expressed as mean ± standard deviation (SD) or median (range). Enumeration data were expressed as frequency or percentage. The clinical characteristics of 103 patients are shown in Table 1.

**TABLE 1:** Clinical characteristics of 103 patients in study.

Characteristics	Value
Sex, *n* (%)	
Male	30 (29.1%)
Female	73 (70.9%)
Age(years, Mean ± SD)	46.96 ± 13.8
Days of hospitalization(days, Mean ± SD)	5.87 ± 1.96
Operation time(minutes, Mean ± SD)	76.36 ± 25.62
Scope of surgery, *n* (%)	
Unilateral	66 (64.1%)
Bilateral	37 (35.9%)
Procedure, *n* (%)	
Thyroid lobectomy	7 (6.8%)
Thyroid lobectomy with CND	49 (47.6%)
Total thyroidectomy	16 (15.5%)
Total thyroidectomy with CND	19 (18.5%)
Laparoscopic unilateral thyroidectomy	3 (2.9%)
Unilateral parathyroidectomy	7 (6.8%)
Bilateral parathyroidectomy	2 (1.9%)

Abbreviation: CND, central neck dissection.

## 3 RESULTS

### 3.1 Computer results

During the ISTR training process, the number of training epoch was set to 72, the backbone network of the model is resnet101 network, and the ImageNet pretraining model was used during the training process. Data augmentation methods including random cropping were used during model training. Among all the data, 79 patients who underwent surgery during the period of August 24, 2021 to July 9, 2022 were used as the internal data set, and the data of the training set and the internal validation set were divided according to 8:2. The internal validation set had a total of 88 images, 115 cases of PGs. The recognition precision and recall of our model on the internal validation set reached 83.5% and 57.8%, respectively. At the same time, the data of 24 patients who underwent surgery during the period of July 9, 2022 to October 18, 2022 were selected as an independent external validation cohort. A precision of 74.5% and a recall of 50.5% were achieved on an independent external validation cohort.

**FIGURE 3:**
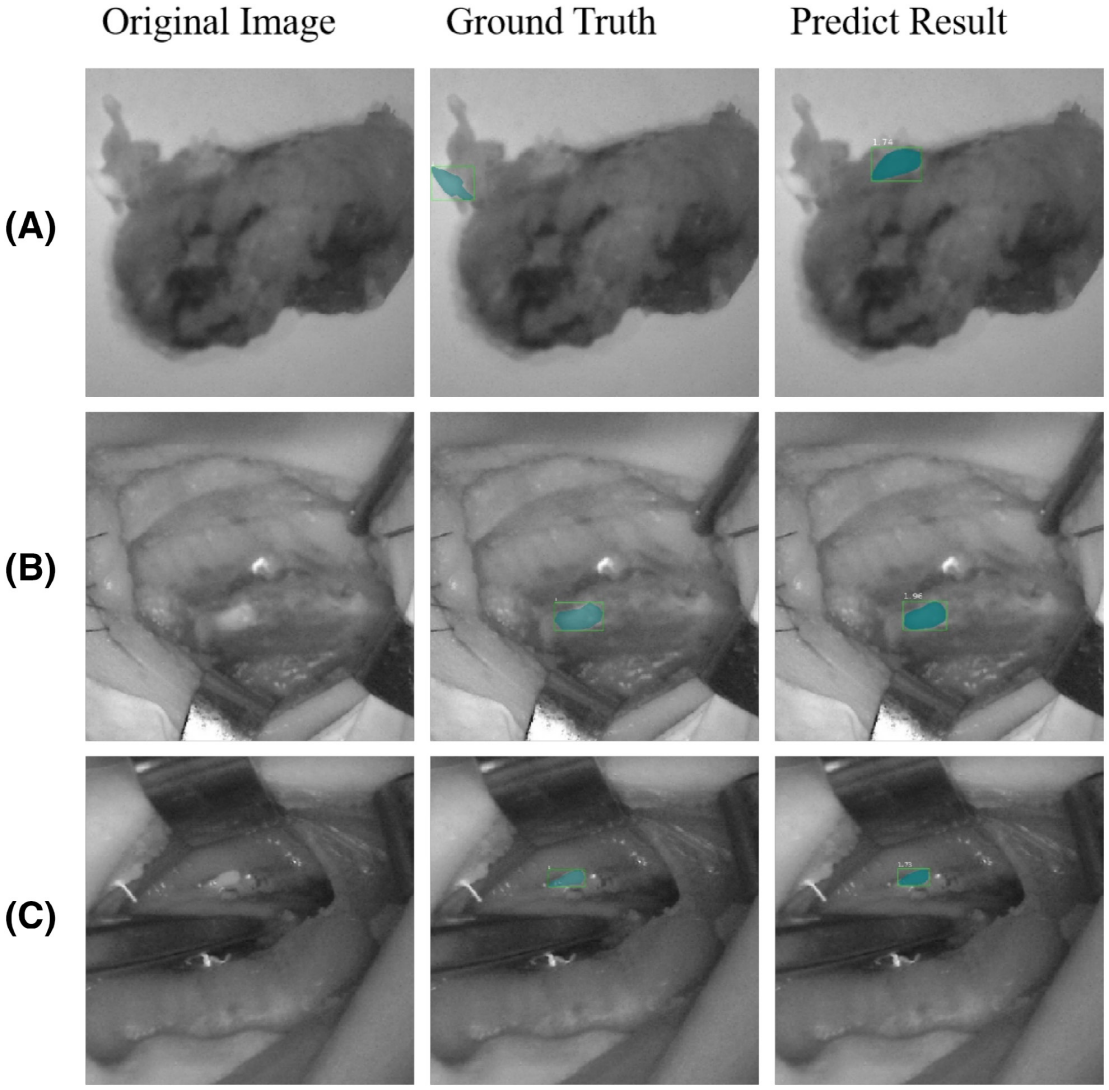
Visualization of predict result of AI model. In group A, AI model incorrectly identified the thyroid nodule with weak fluorescence. In group B and C, AI model could accurately predict the location and shape of PG while not influenced by false positive tissues (scab of energy device in group B and sutures knot in group C).

Since in the research, the data set used NIFI images as the data source, in order to restore the working scene of NIFI as much as possible, all the images in the data set retained a large viewing angle. Since the size of PG was smaller than the surgical field of view, it was a small target in the field of object detection. The model achieved an average precision of 44.0% on small objects. Some visualization of AI model prediction results are shown in Figure 3. Origin image represented original photo, ground truth represented the shape and location of PGs in origin image, predict result represented the prediction result of our AI model. Comparison details are shown in Table 2. Some visualization results are shown in Figure 3.

**TABLE 2:** Different AI model results of parathyroid detection.

Model	Precision	Recall	F1	APs
ISTR	82.2%	56.5%	67.0%	40.8%
ISTR‐R101	83.5%	57.8%	68.3%	44.0%
Mask RCNN	72.5%	39.5%	51.1%	35.8%
SparseRCNN	81.9%	60.0%	69.3%	42.3%

### 3.2 Statistics results

There were 44 patients in the internal validation set with 88 NIFI images, including 29 for stage I, 45 for stage II, and 14 for stage III, 115 PGs in total. The recognition rate of this AI model was 85.2% (98/115). For the same NIFI images, the recognition rate was 70.4% (81/115) for junior surgeon, 73.0% (84/115) for intermediate surgeon, and 83.5% (96/115) for senior surgeon. The recognition rate of AI model was significantly higher than that of junior surgeon (*χ*2 = 9.48, *p* = 0.002) and intermediate surgeon (*χ*2 = 4.69, *p* = 0.029). However, there was no significant difference in recognition rate between AI model and senior surgeon (*χ*2 = 0.06, *p* = 0.804).

There were 24 patients in the external validation cohort with 71 NIFI images, including 18 for stage I, 29 for stage II, and 24 for stage III, 91 PGs in total. The recognition rate of AI model was 82.4% (75/91). For the same NIFI images, the recognition rate was 71.4% (65/91) for junior surgeon, 79.1% (72/91) for intermediate surgeon, and 80.2% (73/91) for senior surgeon. The recognition rate of AI model was significantly higher than that of junior surgeon (*χ*2 = 4.0, *p* = 0.043). However, there was no statistically significant difference between AI model and intermediate surgeon (*χ*2 = 0.31, *p* = 0.581) and senior surgeon (*χ*2 = 0.10, *p* = 0.754).

A total of 234 PGs were detected in 103 patients, of which 190 were detected by white light and 221 were detected by NIFI device. The sensitivity, specificity and accuracy of NIFI to predict PGs were 94.6%, 71.6%, and 87.1%.

The clinical characteristics and PTH levels in different periods of 35 patients underwent bilateral thyroidectomy are shown in Table 3. On the first postoperative day, 48.6% (17/35) of the patients had PTH levels below the lower limit of normal, while 17.1% (6/35) exhibited symptomatic hypocalcemia. After 1 month, PTH levels returned to normal for 14 patients, with only one patient exhibited symptomatic hypocalcemia. During the 3‐month and 6‐month follow‐up assessments, one patient progressed to permanent hypoparathyroidism with symptomatic hypocalcemia. The results are shown in Table 4.

**TABLE 3:** Clinical characteristics of 35 patients underwent bilateral thyroidectomy in study.

Characteristics	Value
Sex, *n* (%)	
Male	9(25.7%)
Female	26 (74.3%)
PTH levels(pg/ml, Mean ± SD)	
Before surgery	43.39 ± 20.17
Postoperative day 1	17.89 ± 11.26
Postoperative 1 month	33.15 ± 19.42
Postoperative 3 months	35.05 ± 15.20
Postoperative 6 months	37.14 ± 15.60

**TABLE 4:** Incidence of hypoparathyroidism and symptomatic hypocalcemia of 35 patients.

	Hypoparathyroidism	Symptomatic hypocalcemia
Postoperative day 1, *n* (%)	17 (48.6%)	6 (17.1%)
Postoperative 1 month, *n* (%)	3 (8.6%)	1 (2.9%)
Postoperative 3 months, *n* (%)	1 (2.9%)	1 (2.9%)
Postoperative 6 months, *n* (%)	1 (2.9%)	1 (2.9%)

### 3.3 False positive analysis

In internal validation cohort, out of 88 NIFI images, 21.6% (19/88) had false positive tissues. 9.1% (8/88) of the images were incorrectly identified as existing PGs by junior surgeon compared to 5.7% (5/88) by the intermediate surgeon and 12.5% (11/88) for senior surgeon. Similarly, 12.5% (11/88) of the images were incorrectly identified as existing PGs by AI model. The false positive tissues identified by different groups are shown in the Table 5.

**TABLE 5:** The false positive tissues identified by different groups for internal validation cohort.

False positive tissue	AI model	Junior surgeon	Intermediate surgeon	Senior surgeon	NIFI images
Deep adipose tissue	6	5	2	6	10
Shallow adipose tissue	2	0	0	0	0
Thyroid nodules	1	2	1	4	6
Muscle	1	0	0	1	0
Sutures knot	1	1	1	0	2
Scab of energy devices	0	0	1	0	1

In external validation cohort, out of 71 NIFI images, 33.8% (24/71) had false positive tissues. 2.8% (2/71) of the images were incorrectly identified as existing PGs by junior surgeon compared to 5.6% (4/71) by intermediate surgeon. However, 11.3% (8/71) of the images were incorrectly identified as existing PGs by AI model. The false positive tissues identified by the different groups are shown in the Table 6.

**TABLE 6:** The false positive tissues identified by the different groups for external validation cohort.

False positive tissue	AI model	junior surgeon	intermediate surgeon	senior surgeon	NIFI images
Deep adipose tissue	3	1	4	3	10
Shallow adipose tissue	2	1	0	0	1
Reflective Spot	2	0	0	0	2
Sutures knot	1	0	0	0	2
Thyroid nodules	0	0	0	0	8
Muscle	0	0	0	0	1

## 4 DISCUSSION

PG is the smallest endocrine organ in body. Because of fragility of PG and similarity of other tissues, it is highly susceptible to injury. The accuracy of naked eye identification of PGs by surgeons with different experience have been reported to be 61%–93.6%, while 5%–22% of patients have one or more PGs unintentionally removed during thyroid surgery.^16^, ^17^, ^18^ Therefore, the rapid identification and precise protection of PGs in thyroid surgery should be paid attention by surgeons.

In 2014, McWade et al. developed the first NIFI device to detect PGs.^19^ The autofluorescence intensity of PGs is 2–6 times higher than that of thyroid, surrounding tissues and background, distinguishing PGs from other tissues. However, it was found that there are some conditions that affect the accuracy of NIFI devices for detecting PGs. For example, false positive occur when detecting brown adipose tissue, lymph nodes, and thyroid nodules.^16^ Our result indicates that the false positive specimens of NIFI in thyroid nodules, adipose tissues, muscle and sutures knot, where bright spots could be found. These tissues were verified as non‐PG by ICGT. However, the large number of false‐positive specimens affects the surgeon's judgment of PGs, and ICGT adds additional time to procedure and financial stress to the patients.

Recent AI advancements in medical applications showcase its superior memory and efficiency compared to human practitioners. This positions AI‐assisted diagnosis for significant and promising development. Wang et al. have confirmed the capability of AI technology for the automatic localization and recognition of PGs in endoscopic surgery.^20^ SN Avci et al. used object detection technology to recognize PGs in NIFI images, surpassing visual recognition by surgeons.^21^ Önder et al. applied a deep‐learning method, Unet, for the segmentation of PGs in CT images.^22^ Zhu et al. introduced an AI method for the semantic segmentation of the entire volume of head and neck anatomy in CT images, effectively distinguishing pixel categories in CT images.^23^ All mentioned methods focus on detecting or segmenting organs or tissues in the head and neck region, primarily in traditional medical imaging such as CT. Our approach uniquely combines AI with NIFI technology for improved PG detection. In our study, we employed Hu et al.'s instance segmentation model designed for real images, adapting it to the challenges of segmenting PGs in NIFI images.^13^ This marks the inaugural application of instance segmentation techniques in AI for PGs recognition in NIFI.

Visualization is a very important part of AI research in clinic. Surgeons of different experiences were enrolled to identify PGs in our study. Although the identification and depiction of PGs by different surgeons introduce an inherent subjective element, criteria for depicting outline of PGs was based on the anatomical location and morphology specified in the authoritative guideline, serving as the reference points.^24^ AI model is able to assist surgeons to some extent in finding and identifying false positive PGs in NIFI images. Forty‐three NIFI images (27.0%, 43/159) in the validation set had false positive tissues, mainly adipose tissues, thyroid nodules, sutures knot and scab of surgical energy devices. We further explore whether AI models could enhance the identification accuracy of false positive and negative tissues of NIFI. Among the 24 NIFI images with false‐positive tissues and three NIFI images with false‐negative tissues, the accurate identification of AI model was 66.7% (16/24), and with false‐negative rate was 66.7% (2/3). These findings suggest that our AI model has the potential to assist NIFI to reduce interference. However, the false‐positive rate of the AI model was higher than the surgeon's recognition for either internal or external validation sets. When surgeons lacked confidence in the recognition of PGs, they failed to circle PGs in the images, which reduced the false‐positive rate and reduced the correct recognition rate. Meanwhile, the AI model circled other suspected tissues to reduce the missed PGs. We found that some of them were shallow adipose tissue, thread knots and reflective spots in NIFI images, which could be easily excluded by surgeon. Therefore, the AI model can assist surgeon in finding hard‐to‐find PGs.

Our model provided a description of the PGs contour while identifying the bounding box position. To the best of our knowledge, our study is the first deep learning model applied in PG recognition and segmentation in NIFI images during thyroidectomy and parathyroidectomy. Our methodology enhances the evaluation of spatial relationships between PGs and surrounding vasculature and tissues, providing surgeons with improved identification and anatomical visualization. The recognition accuracy of the AI model is similar to that of senior surgeon, which is much greater than that of junior surgeon. In the internal and external validation set, the recognition rate of AI model was significantly higher than that of junior surgeon and that of intermediate surgeon in the former validation set. In the external validation set, there was no statistically significant difference between AI model and intermediate or senior surgeons, which means that AI model had similar recognition ability to professional surgeons. Moreover, the recognition accuracy of AI model was higher than that of surgeons with different experience. AI model was able to detect and give more accurate judgments on ambiguous fluorescent tissues. These advantages help us to accurately identify PG intraoperatively.

In addition, AI model helps surgeons who have just entered the field familiar with the shape and location of PG, and assist in identifying the correct PGs. Furthermore, the provided contour masks can reflect the integrity of PG to avoid damage itself and protect surrounding blood supply when dissecting the membrane of PGs and central neck lymph nodes, and provides better visual feedback.

Our study, though impactful, is limited by its single‐center design and a modest patient cohort, necessitating broader datasets and multi‐center validations to enhance AI model recognition rates. PTH levels was confined to 35 patients from our observational data, underscoring the need for a randomized controlled trial to thoroughly assess AI‐assisted recognition in NIFI detection. While our AI method excels in PGs segmentation within NIFI, the imperative transition towards video segmentation is evident for nuanced analysis, elevating its clinical significance.

## 5 CONCLUSIONS

This is the first deep learning model for recognition and segmentation of PGs in NIFI images. The recognition precision and recall of the AI model reached 83.5% and 57.8% on the internal validation set. The recognition rate of AI model was 85.2% and 82.4% in internal and external validation set under visualization, similar to that of intermediate and senior surgeons. Our model can accurately label PGs while delineating the outline of PGs, so as to better observe and protect during surgery. At the same time, the recognition accuracy of the AI model in NIFI images is higher than that of junior and intermediate surgeon, and similar to that of senior surgeon. The AI model reduces the interference of false positive tissues in NIFI images, and assists surgeons to further identify PGs.

## AUTHOR CONTRIBUTIONS

**Fan Yu:** Data curation (equal); formal analysis (equal); writing – original draft (equal); writing – review and editing (equal). **Tian Sang:** Conceptualization (equal); data curation (equal); methodology (equal); software (equal); writing – original draft (equal); writing – review and editing (equal). **Jie Kang:** Validation (equal); visualization (equal). **XianZhao Deng:** Validation (equal); visualization (equal). **Bomin Guo:** Validation (equal); visualization (equal). **Hangzhou Yang:** Validation (equal); visualization (equal). **Xiaoyi Chen:** Funding acquisition (equal). **Youben Fan:** Funding acquisition (equal). **Xuehai Ding:** Project administration (equal); supervision (equal); writing – review and editing (equal). **Bo Wu:** Supervision (lead); validation (equal).

## CONFLICT OF INTEREST STATEMENT

All authors do not have any conflicts of interest regarding this study.

## Data Availability

The data are not publicly available due to privacy or ethical restrictions.
